# Editorial: Animal welfare, Volume I: Animal welfare in aquaculture - Physiological basis and recent findings

**DOI:** 10.3389/fphys.2022.1097913

**Published:** 2022-11-25

**Authors:** M. Herrera, B. Costas, E. Gisbert

**Affiliations:** ^1^ Department of Agroforestry Sciences, University of Huelva, Huelva, Spain; ^2^ Centro Interdisciplinar de Investigação Marinha e Ambiental, Universidade do Porto (CIIMAR), Matosinhos, Portugal; ^3^ Instituto de Ciências Biomédicas Abel Salazar (ICBAS), Universidade do Porto, Porto, Portugal; ^4^ Aquaculture Program, Institut de Recerca i Tecnología Agroalimentaries (IRTA), La Rápita, Spain

**Keywords:** aquaculture, stress, welfare, physiology, fish

Since animal welfare is a multidisciplinary scientific discipline, the papers published in this special issue are based on diverse subjects, such as feeding and nutrition, behavior, immunology, reproduction, pathology, endocrinology, and neuroendocrinology. The studies targeting fish species have been the most frequent, being salmonids the main group. Nevertheless, only one out of the thirteen published articles deals with an invertebrate species, the lobster (*Homarus americanus*), which highlights the recent interest for crustaceans’ welfare. The results derived from these works state objectively the importance of animal welfare in both physiological and behavioral traits and responses, as well as in zootechnical parameters of interest for farmers.

The feeding and nutrition studies linked to fish welfare are an important group in the present topic. In particular, Salamanca et al. aimed to evaluate the effects of functional feeds supplemented with phenylalanine and tyrosine on the endocrine condition and physiological status of gilthead seabream (*Sparus aurata*) exposed to chronic stress. These authors reported that fish fed functional supplemented diets showed a reduction in various stress markers and physiological parameters. Additionally, they concluded that the stress condition favored a mobilization of amino acids towards the tissues, especially in supplemented diets, so this excess of amino acids could be used as an energy substrate to cope with stress, which is of special relevance for aquafeed manufacturers and fish farmers. The second study in this special issue dealing with nutrition was focused on probiotics. Amenyogbe et al. aimed to select potential probiotic bacteria from cobia (*Rachycentron canadum*) gut to be used as feed additive as a prophylactic nutritional strategy. Those authors isolated three indigenous bacteria with potential probiotic effects: *Bacillus* sp., *Pantoea agglomerans* and *B. cereus*. These bacteria demonstrated antagonistic effects on the growth of pathogenic *Vibrio alginolyticus*, *V. harveyi*, *Streptococcus iniae*, and *S. agalactiae*, etiological agents of vibriosis and streptococcosis. Such effect against pathogenic bacteria was achieved by their capacity of producing antibiotic compounds, hence authors suggested that their inclusion in functional feeds could prevent or control disease outbreaks instead of using antibiotics or chemicals. Furthermore, Pelusio et al. and Sundell et al. have published a more classical nutritional study, based on fishmeal (FM) and oil (FO), and dietary fatty acids contents. In brief, Pelusio et al. focused on investigating the interaction between very low dietary levels of FM and FO and animal welfare in gilthead seabream (*Sparus aurata*) when exposed to a crowding stress challenge. For evaluating the effect of dietary regimes on fish condition, authors analyzed a wide range of parameters like stress and metabolic biomarkers in plasma, humoral immunity biomarkers in skin mucus and stress, and immune gene expression markers from the brain and the head-kidney. Overall, this study concluded that low FM (10%) and FO (3%) levels might compromise stress and immune responses, as well as growth performance in farmed fish. Sundell et al. carried on a more specific study, at intestinal level, on dietary omega-3 fatty acids in Atlantic salmon (*Salmo salar*) smolts. Specifically, those authors stated that low levels of omega-3 in the diet did not affect osmoregulation but decreased growth and changed the fatty acid composition of the proximal intestine. Additionally, those levels also reduced omega-3 content of the intestinal epithelial membranes as well as in impaired intestinal barrier function and decreased TEP (transepithelial potential difference) in the proximal intestine. Consequently, the authors concluded that low levels of omega-3 in the diet impaired the intestinal functions, which might reduce fish health and welfare.

The study of fish behavior in order to get a better understanding of fish welfare is increasingly gaining interest. In fact, it has been reported that the analysis of fish behavior coupled with personality studies is a good tool for proper understanding and improvement of fish welfare. In the present research topic, Li et al. evaluated the background color preference of the lined seahorse (*Hippocampus erectus*) and its relationship with personality (bold vs*.* shy) by assessing fish preference for different colored tank compartments (white, red, green, black, yellow, and blue backgrounds). Authors found that the lined seahorse showed a general preference for white and blue backgrounds, while they avoided black and red backgrounds. Therefore, this study recommended to use white and blue background colors for culturing this seahorse species, whereas they also proposed that it was able to assess the personality (bold-shy) of fish by means of their background color preference. A similar study was carried on by Yi et al., who researched on the effects of the light spectra on the GIFT tilapia (*Oreochromis niloticus*) welfare. Thanks to a transcriptomic analysis, the authors stated that different light spectra could regulate signaling pathway associated with the tilapia juveniles growth, stress, and behavior. Specifically, that work demonstrated that white and red lights affected positively the growth performance, meanwhile yellow light enhanced fish stress and aggressive behaviors. Following with the study of light as key factor in fish physiology, behavior and welfare, Frau et al. analyzed the ontogeny of six visual photopigments in Senegalese sole (*Solea senegalensis*) specimens at different ages. Such study using qPCR and *in situ* hybridization techniques was conducted in animals reared under different light conditions (light-dark cycles of white, blue, red, and continuous white lights) to understand how visual photoreceptors adapted to different light conditions. The authors concluded that visual opsin transcript levels changed during ontogeny depending on developmental stages and light regime and recommended using blue/green lights as an alternative to white lights in sole rearing protocols during early stages.

Probably, cortisol is the stress indicator which has been more used and studied in fish welfare studies. The works in the present topic do not suppose an exception and some works on cortisol dynamics and analysis have been published. It is known that the stress response in fish modulate the immune system due to systemic cortisol actions, including responses at mucosal tissues level. In this sense, Vallejos-Vidal et al. aimed to better understand whether cortisol can also play a modulatory role on the mucosal transcriptomic profile through its presence in the mucosal tissue surface of two important farmed species, the rainbow trout (*Oncorhynchus mykiss*) and the gilthead seabream. Therefore, an *ex-vivo* study was conducted to evaluate in a time-course fashion the effect of cortisol on selected immune- and stress-related genes. Those authors concluded that the quick modulation of the gene expression during the first 24 h after the exposure to a stressor challenge reported in previous studies, is probably coordinated and mediated through a systemic-dependent mechanism but not through a peripheral/local response on mucosal tissue surfaces. A more innovative approach to the analysis of cortisol was described by Höglund et al., who assessed stress resilience in Atlantic salmon after smolt transportation through waterborne cortisol. Those authors suggested that, together with behavioral responses to feed, water cortisol measurements are promising non-invasive indicators of adaptive processes associated with stress resilience in recirculating aquaculture systems (RAS). Additionally, that method was suggested to be used for evaluating the impact of potential stressors, such as compromised water quality, crowding and handling, on fish welfare. However, they also reported that water cortisol concentrations and cortisol release rate are sensitive to system (RAS) perturbations. Besides cortisol, the research on other hormones or neurotransmitters for evaluating stress response at endocrine and neuronal level is also enhancing. In this context, Staven et al. researched the responses of lumpfish (*Cyclopterus lumpus*) to the presence of Atlantic salmon or salmon sensory cues. They analyzed the catecholamine and cortisol levels in plasma and tissues, and other parameters in lumpfish cohabiting with Atlantic salmons. Interaction with live salmon induced alterations in the brain of the lumpfish, which revealed reduced levels of brain catecholamines, namely norepinephrine and dopamine. However, health scores and skin coloration remained unaltered. Based on these results, authors concluded that lumpfish were not negatively impacted by cohabitation or exposure to salmon cues, which suggested that welfare disruption in commercial production is probably related to a combination of Atlantic salmon exposure with other stressors.

Recently, the importance of fish skin and associated mucus for fish health and welfare has been subject of interest in many works. In this sense, Doyle et al. looked into the transport and barrier functions in rainbow trout skin. They assessed the contribution of the different skin layers to barrier function, determined the effect of salinity, and tested for possible active transport mechanisms, stating that the epidermis was the diffusion barrier in the skin, with the scales and dermis playing a negligible role. The study also reported that environmental salinity affects significatively the epidermal barrier function, demonstrating that freshwater exposure derived in a high reduction of epithelial permeability. Nevertheless, regards active transport, they concluded that further research is required to determine if skin V-ATPase contributes significantly to overall ion homeostasis in rainbow trout, or whether it plays a more specialized role.

The only work on invertebrates published in this Research Topic deals with the involvement of crustacean hyperglycemic hormones (CHHs) in the reproductive cycle of the female lobster (Wang et al.). This basic research showed that HaCHH-A and HaCHH-B transcripts are derived from two different 4-exon CHH genes, and each gene can produce different but larger transcript variants (i.e., sHaCHH-A and sHaCHH-B) mainly in different non-eyestalk tissues of the females. Results showed that recombinant protein for sHaCHH-A and sHaCHH-B inhibited vitellogenin gene expression whereas dsRNA for sHaCHH-A and sHaCHH-B inhibited the expression of vitellogenin gene *in vitro*. The authors concluded their findings can provide insights for the development of techniques to induce gonad development without the use of the eyestalk ablation operation, improving the animal welfare.

The successful application of findings coming from research is the last and crucial step for an effective research activity. In this sense, a technical and general approach to the study of fish behavior and welfare was performed by Calduch-Giner et al., who revised the use of a new bio-logger (AEFishBIT) that allows the simultaneous monitoring of swimming activity and ventilation rates under steady and unsteady swimming conditions. The proof of concept of such technology was validated under different tools like video recording, exercise tests in swim tunnel respirometers, and differential operculum and body tail movements across fish species with differences in swimming capabilities. Authors concluded their review recommending the incorporation of behavioral variables in fish as a routine procedure of academia researchers as well as fish farmers, by the application of bio-loggers that accurately measure their swimming activity among other physiological variables.

To conclude, topic editors hope that above mentioned works can contribute to a deeper knowledge on animal welfare in aquaculture, and thank and congratulate authors for their excellent findings presented here. In this sense, topic editors encourage them to follow researching within this topic.

